# Silicon as a Strategy to Mitigate Abiotic Stresses and Improve Physiological Performance and Grain Yield of Maize Grown Under Tropical Climate Conditions

**DOI:** 10.3390/plants14172755

**Published:** 2025-09-03

**Authors:** Mateus de Leles Lima, Rilner Alves Flores, Maxuel Fellipe Nunes Xavier, Renato Gomide de Sousa, Derblai Casaroli, Felipe Puff Dapper, Frank Freire Capuchinho, Glenio Guimarães Santos, Klaus de Oliveira Abdala, Letusa Momesso

**Affiliations:** 1 Federal Institute of Science and Technology of Roraima (IFRR), Amajari 69343-000, RR, Brazil; mateus.lima@ifrr.edu.br; 2 School of Agronomy, Federal University of Goiás (UFG), Goiânia 74690-900, GO, Brazil; maxuelfellipe90@gmail.com (M.F.N.X.); gomide@discente.ufg.br (R.G.d.S.); derblai@ufg.br (D.C.); felipe.pdapper@gmail.com (F.P.D.); frankfreirec@gmail.com (F.F.C.); gleniogm@ufg.br (G.G.S.); agroklaus@ufg.br (K.d.O.A.); letusa.momesso@ufg.br (L.M.)

**Keywords:** foliar fertilization, gas exchange, hydric deficit, root development, silicon fertilization, *Zea mays* L.

## Abstract

Although the beneficial effects of silicon on plant resistance to biotic and abiotic stresses are recognized, there is a lack of knowledge regarding its application in field conditions and its direct impact on physiological metabolism, root development, and, most importantly, the economic return of corn production in tropical regions. This study is justified by the need to quantify the effects of foliar silicon application on these variables, providing a scientific and economic basis for optimizing corn productivity and profitability in tropical environments. The objective of this study was to evaluate the effect of silicon on physiological metabolism, root system development, grain yield, and the potential economic return of maize production in a tropical region. The study was conducted under field conditions in two growing seasons (2020 and 2021), using a randomized block design in a 2 × 5 factorial arrangement with four replications. The first factor consisted of the maize growing seasons, and the second factor was foliar silicon fertilization (0 (control), 150, 300, 450, and 600 g ha^−1^). Foliar fertilization with silicon at a dose of 150 g ha^−1^ increases transpiration rate by up to 9%, net photosynthetic rate by 13%, and grain yield of maize by 10% after two growing seasons, regardless of the water deficit experienced during the crop cycle. At this dose, silicon application is economically viable, yielding the highest differential profit (USD 97.11 ha^−1^). In conclusion, foliar fertilization with silicon is an agronomically and economically viable strategy for efficient maize grain production during the second growing season in tropical regions.

## 1. Introduction

Climate change represents a serious global issue, affecting the entire spectrum of living organisms, including cultivated plants [1]. Climate conditions can determine the rate of plant growth, which appears to be altered due to the pressure of global warming [2]. Climate change leads to uneven precipitation patterns (with prolonged droughts often followed by intense rainfall) and exposes plants to stress from high temperatures combined with increased ultraviolet (UV) radiation [3].

Agriculture is a key sector of the global economy and is highly dependent on climate conditions [1]. One of the greatest challenges of the current century is to ensure high crop yields in the context of climate change while simultaneously preserving natural resources [4]. Among the various types of food commodities, such as crops, livestock, fisheries, and aquaculture, crops are by far the most affected by climate change, with yield losses reaching up to 50% [5]. In this context, drought is one of the most severe abiotic stresses limiting crop productivity worldwide and poses a serious threat to agricultural sustainability in tropical regions [6].

This situation is particularly relevant in the Brazilian Cerrado, one of the world’s most important grain-producing regions, where the expansion of maize cultivation, especially in the second crop, has been threatened by shorter rainy seasons and increasing water deficits. Multimodel evaluations indicate that maize productivity consistently declines with rising temperatures, with average reductions of approximately 0.5 t ha^−1^ for every 1 °C increase, depending on the cultivar and management practices [7]. Similarly, regional simulations using the Multidisciplinary Simulator for Standard Cultures (STICS) model for the Cerrado highlight that future warming and altered rainfall patterns will likely reduce water availability and shorten the growing cycle, intensifying stress during critical phenological stages [8]. Taken together, these results highlight that climate change poses a tangible threat to maize production in Goiás and the Cerrado, an important tropical agricultural frontier.

Maize (*Zea mays* L.) is one of the most important food crops worldwide, playing a direct role in global food security and serving as a major cereal for both human and animal consumption [9]. Currently, Brazil is the third-largest maize producer globally [10,11], with an annual production of 122 million tons of grain cultivated over an area of 21.1 million hectares and an average yield of 5758 kg ha^−1^ in the 2023/2024 growing season. Of this total, 80% was produced during the second growing season, typically cultivated between February and September [12].

Although second-season maize cultivation is a common practice in Brazil, average yields remain below the crop potential, mainly due to abiotic factors such as high temperatures and water scarcity, as well as limited input investment stemming from the increased climatic risks [13,14]. In Brazil, the second harvest corresponds to the cultivation carried out immediately after the harvest of the main summer crop, taking advantage of the same agricultural area, with maize being the main crop established during this period. This system is highly relevant for tropical agricultural regions around the world, as it increases the supply of grains, optimizes the use of soil and climate resources, and contributes to the sustainability of agricultural production.

Although Si is the second most abundant element in the Earth’s crust, with approximately 28% in the form of silicon dioxide (SiO_2_), following oxygen (O_2_) [15], it is predominantly found in a non-labile form for plants (SiO_2_). This is because its absorbable form is monosilicic acid (H_4_SiO_4_) [16], which occurs in soils at low concentrations, typically ranging from 0.1 to 0.6 nM, and can be taken up by plant roots when the soil pH is below 9 [17].

In tropical regions, silicon (Si) can be an alternative to mitigate the deleterious effects caused by biotic and abiotic stresses [18]. In this context, Si can modulate gas exchange processes (i.e., internal CO_2_ concentration, stomatal conductance, net photosynthetic rate, and transpiration) and pigment production (i.e., chlorophyll) [19,20,21], mitigate abiotic and biotic stresses [14,22], and promote gains in plant growth, productivity, and product quality [19,23,24].

In this context, the use of Si in plants may enhance plant–environment interactions due to its capacity to improve plant tolerance to environmental stresses [23,25,26,27]. According to the literature, studies on Si supply in maize have demonstrated its effectiveness through various mechanisms, including enhanced resistance to *Stenocarpella macrospora* leaf spot [28] and anthracnose (*Colletotrichum graminicola*) [29], reduced water demand under drought conditions [6], alleviation of potassium (K) [30] and magnesium (Mg) deficiencies [31], improved manganese use efficiency [32], and mitigation of toxicity caused by ammonium (NH_3_) [33], cadmium [34], zinc [35], salinity [36], and UV radiation [37]. However, these studies were conducted under stress-inducing conditions, and there is a scarcity of research focused on the effects of Si on maize under field conditions. This highlights both the limited understanding and the promising potential of foliar Si application in this crop, emphasizing the need to assess its agronomic performance and economic viability under real-world production systems.

In this regard, with an emphasis on advancing research related to the effects and development of application recommendations for Si in annual crops, studies have been conducted on members of the *Poaceae* family under non-stress conditions, such as sorghum (*Sorghum bicolor* L.) [24,38] and wheat (*Triticum aestivum* L.) [39,40], yielding promising results. These findings underscore the scarcity of studies involving the application of soluble Si sources—such as K and copper (Cu) silicate stabilized with sorbitol—in maize crops under field conditions. On this basis, the following hypothesis was proposed: foliar application of Si enhances its absorption by plants, modulates gas exchange, improves developmental parameters (root system and grain formation), and promotes increases in yield and differential profit (economic analysis). Therefore, the objective of this study was to evaluate the effect of foliar Si fertilization on plant physiology, root development, grain yield, and differential profit in second-season maize grown under tropical climate conditions.

## 2. Results

**Figure 1:**
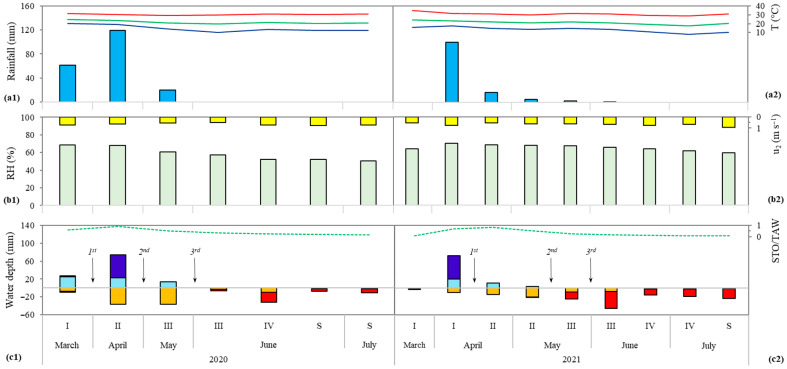
Accumulated rainfall (blue column), air temperature (T, °C) maximum (red line), average (green line), and minimum (blue line) (**a**); relative humidity (RH, %) (green column) and wind speed (u_2_, m s^−1^) (yellow column) (**b**); water excess (dark blue column) and deficit (dark red column), water withdrawal (light blue column), replacement (orange column), and relative soil water storage (STO/TAW) (**c**); identified for each phenological stage (I: establishment; II: vegetative; III: flowering; IV: yield formation; S: ripening) of maize, month (from March to July) of the year and year of cultivation, in harvest 2020 (**1**) and 2021 (**2**). 1st, 2nd, and 3rd—first, second and third foliar application of silicon, respectively.

The accumulated rainfall (R) during the maize cycle (2020) was 201.30 mm, with 59% concentrated in Phase II, 31% in Phase I, and 10% in Phase III. Additionally, the mean maximum (T_M_), average (T_a_), and minimum (T_m_) air temperatures during the cycle were 30.1, 21.8, and 15.6 °C, respectively (Figure 1(a1)). The mean relative humidity (RH) and wind speed (u_2_) were 61 ± 6.21% and 0.64 ± 0.10 m s^−1^, respectively (Figure 1(b1)). The accumulated water surplus (102.08 mm per cycle) was slightly below the deficit (108.38 mm per cycle), with the greatest surplus recorded in Phases II and I (74.50 and 27.58 mm, respectively) and the greatest deficit in Phase III (40.87 mm). Furthermore, the average soil moisture during the cycle was just over half of the available water capacity (AWC), approximately 52%, with average values of 88% in Phase II, 58% in Phase I, 48% in Phase III, 24% in Phase IV, and 17% in the Senescence phase (S) (Figure 1(c1)).

During the 2021 growing season, the accumulated rainfall (R) was lower than that of the previous season (2020), totaling approximately 125.20 mm, with 80% and 17% of this amount occurring in phenological stages I and II, respectively, summing to 121.80 mm. The mean maximum (T_M_), average (T_a_), and minimum (T_m_) air temperatures during the cycle were 30.7, 20.9, and 13.1 °C, respectively (Figure 1(a2)). Mean relative humidity (RH) and wind speed (u_2_) were 66 ± 2.70% and 0.68 ± 0.10 m s^−1^, respectively (Figure 1(b2)). Similar to the 2020 season, a higher accumulated water deficit (159.47 mm per cycle) was observed in 2021 compared to the surplus (75.63 mm per cycle). The surplus was concentrated in stages I (71.89 mm) and II (3.73 mm), whereas deficits were recorded in stages III (69.23 mm) and IV (34.96 mm), with the remaining stages (I, II, and S) exhibiting deficits not exceeding 24.10 mm. The average soil moisture during the cycle was lower than in the previous season, representing 33% of the available water capacity (AWC), with higher values of 63%, 61%, and 18% of AWC recorded in stages II, I, and III, respectively, and lower values observed in stages IV (8.14% of AWC) and S (5.79% of AWC) (Figure 1(c2)).

**Figure 2:**
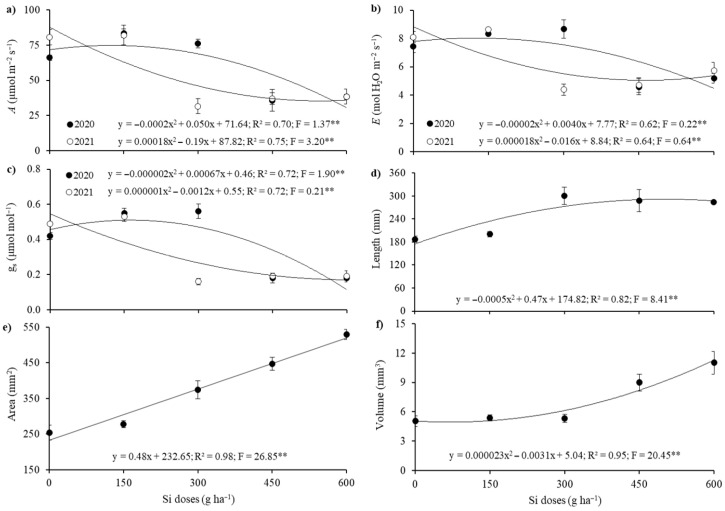
Unfolding of the rate of net photosynthesis (*A*) (**a**), transpiration (*E*) (**b**), and stomatal conductance (g_s_) (**c**), as a function of interactions between the harvests evaluated and foliar application of silicon. Length (**d**), specific surface area (**e**), and volume (**f**) of maize roots, as a function foliar application of silicon. **—significant at 1% probability by the F test.

Net photosynthetic rate (*A*), transpiration rate (*E*), and stomatal conductance (g_s_) were significantly influenced by the interaction between growing seasons and silicon application rates (Table 1). These variables exhibited quadratic responses; in the 2020 season, increases were observed as a function of the applied doses (maximum inflection points: *A* = 74.77 µmol m^−2^ s^−1^ at 125 g ha^−1^ Si; *E* = 7.97 mmol m^−2^ s^−1^ at 100 g ha^−1^ Si; and g_s_ = 0.52 mmol m^−2^ s^−1^ at 168 g ha^−1^ Si). In contrast, the 2021 season showed reductions in the physiological variables (*A*, *E*, and g_s_), with minimum inflection points at the 300 g ha^−1^ Si dose, resulting in decreases of 61%, 46%, and 67%, respectively, compared to the control treatment without silicon fertilization (Figure 2a–c).

**Figure 3:**
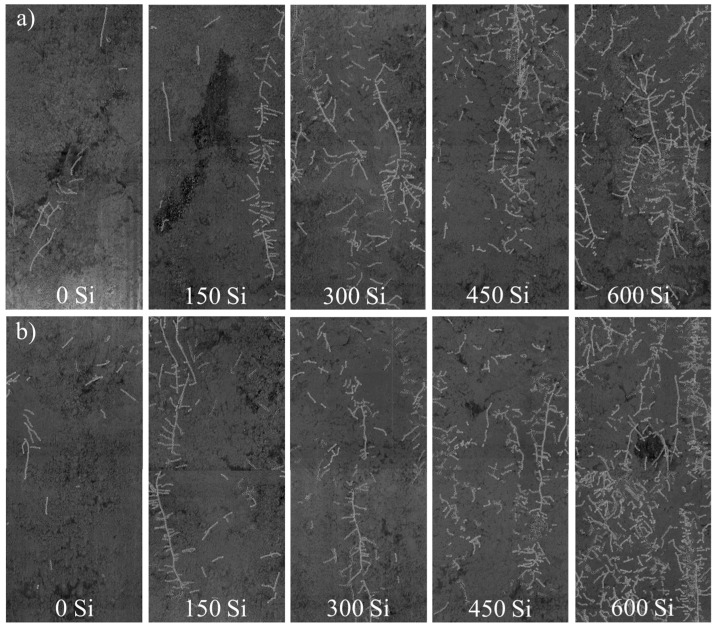
Images of the root system of maize plants, in the 0–0.40 m layer, obtained by the minirhizotron method, as a function of foliar application of Si (0, 150, 300, 450 and 600 g ha^−1^), in the 2020 (**a**) and 2021 (**b**) harvests.

No significant differences were observed in root system development of maize plants between the growing seasons (Table 1). However, foliar fertilization containing silicon promoted increases in root system development across all evaluated variables (root length, specific surface area, and root volume), as shown in Table 1 and illustrated in Figure 3. Root length exhibited a significant quadratic response, increasing up to the dose of 300 g ha^−1^ of Si, reaching 300.62 mm, which corresponds to a 61% increase compared to the treatment without silicon application (Figure 2d). When evaluating the results for the specific root surface area, a significant positive linear trend was observed, with increases up to the highest applied dose, 600 g ha^−1^ of Si, reaching 529.25 mm^2^, representing a 108% increase relative to the control treatment (Figure 2e). Although the total root volume showed a significant decreasing quadratic adjustment, the highest volume (11.02 mm^3^) was observed at the largest Si dose applied, indicating a 119% increase compared to the control treatment (Figure 2f).

**Figure 4:**
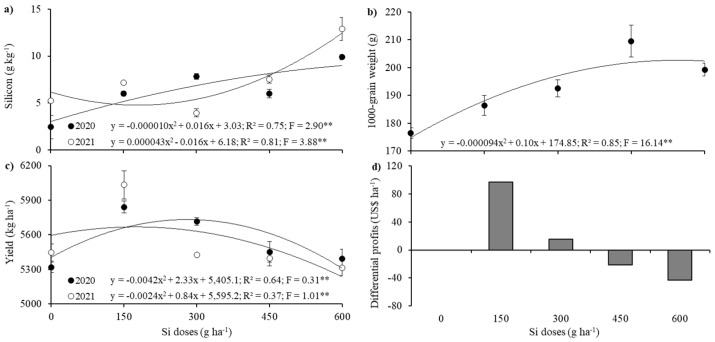
Unfolding of silicon content (**a**) and maize grain yield (**c**) as a function of interactions between the harvests evaluated and foliar application of silicon. Unfolding of 1000-grain weight (**b**) of maize as a function of foliar application of silicon. **—significant at 1% probability by the F test. Economic analysis of the differential profits (DP) of the maize crop as a function of foliar application of Si, in the two harvests (**d**).

Potassium (K) and copper (Cu) contents in the plants showed no significant effects due to growing seasons or silicon application rates, with mean concentrations of 21.43 g kg^−1^ and 21.85 mg kg^−1^, respectively. Silicon content in the plants, however, was influenced by both growing seasons and silicon doses, as shown in Table 2. The interaction between growing seasons and silicon application rates exhibited significant quadratic responses, with the highest Si dose (600 g ha^−1^) resulting in the greatest plant silicon contents of 1.98 and 2.58 g kg^−1^ for the 2020 and 2021 seasons, respectively (Figure 4a). These increases correspond to gains of 304% and 146% compared to the control treatment without silicon addition. Regarding thousand-grain weight (TGW), a significant effect was observed only for the silicon doses applied (Table 2), showing a quadratic response with the highest weight of 209.55 g at 450 g ha^−1^ Si application, representing a 19% increase relative to the control treatment (Figure 4b).

Maize grain yield showed a significant effect between the evaluated growing seasons and silicon application rates, as presented in Table 2. In both seasons, the data exhibited a significant quadratic response, with the highest grain yields obtained at the 150 g ha^−1^ Si dose, corresponding to a 10% increase in productivity compared to the control treatment, approximately 9 bags ha^−1^ (60 kg bags) or 556 kg ha^−1^ (Figure 4c).

Figure 4d displays the results of differential profits relative to the applied treatments. It was observed that only the 150 and 300 g ha^−1^ Si doses resulted in positive differential profits and were therefore economically efficient compared to the control treatment. The treatment with 150 g ha^−1^ Si generated the highest differential profit of USD 97.11 ha^−1^. These results highlight the importance for producers to account for differential profits of various technologies and provide information that enables proper financial management of technological adoption for efficient agricultural input application.

## 3. Discussion

For maize cultivation, the recommended temperature range is between 21 and 28 °C [41,42], which aligns with the average temperatures recorded in the present study: 21.8 °C and 20.9 °C in the 2020 and 2021 growing seasons, respectively (Figure 1(a1,a2)). The maximum tolerable temperature is 35 °C, and the base temperature ranges from 8 to 10 °C. Additionally, the recommended average relative humidity (RH) should be above 70% [43]; however, the averages observed in this study were lower: 61 ± 6.21% and 66 ± 2.70% in the respective seasons (Figure 1(b1,b2)). The water demand throughout the entire maize cycle ranges from 500 to 800 mm, due to maize’s efficiency in water use linked to biomass production, and among cereals, aiming to achieve maximum productive potential [10]. Nevertheless, accumulated rainfall (R) was 201.30 mm and 125.20 mm for 2020 and 2021, respectively (Figure 1(a1,a2)).

Water deficit in maize can cause losses at all growth stages [42], and in the present study, it was more pronounced in stages III and IV in both seasons (2020: 40.90 and 31.81 mm, respectively; 2021: 69.23 and 34.96 mm, respectively; Figure 1(c1,c2), which correspond to flowering (III) and grain formation/filling (IV). A deficit during stage III can lead to the abortion of embryonic sacs in spikelets, desiccation of styles and stigmas (increasing protandry), meiosis disturbances, and pollen grain death, resulting in yield reduction. Conversely, a deficit during stage IV affects plant metabolism (reductions in g_s_ and *A*), consequently decreasing the production of photoassimilates and their translocation to maize grains [42].

However, it is important to emphasize the complexity of quantifying the effects of stress due to low water availability caused by water deficit. In many maize-producing regions, plants experience water deficit, high solar radiation, and extreme temperatures—factors that increase evapotranspiration, exacerbating the deficit’s impact on the plant [44]. Therefore, the use of water balance in field research, following the methodology of Thornthwaite and Mather [45], is feasible, as demonstrated by Xavier et al. [20,46]. Furthermore, the efficiency of silicon (Si) in mitigating the deleterious effects of water deficit can be enhanced through innovative practices, such as using soluble sources of this beneficial element to prepare solutions with concentrations below the Si polymerization threshold (3 mmol L^−1^) [47], as performed in the present study. Thus, the beneficial effects of Si observed on gas exchange parameters possibly mitigated the effects of water stress (Figure 1(c1) and Figure 2(c2)).

The efficiency of foliar silicon (Si) application depends on the Si source and applied dose [48], since Si supply to crops has favored development under stress conditions [49]. Crops are occasionally exposed to different types of stress (biotic or abiotic), which can induce physiological disorders [50]. Thus, Si application to plants can alleviate such deleterious effects by reducing cell membrane degradation, which helps maintain the integrity of photosynthetic pigments, promoting higher production of chlorophyll and carotenoids—antioxidants that reduce degradation of various organic compounds in the plant, including chlorophyll—and oxidative stress [51,52,53,54]. Therefore, the foliar supply technique of soluble Si in the form of SiKCu may have mitigated the deleterious effects described by Cruz et al. [42] and Souza and Barbosa [44] for maize, based on results from the studied growing seasons, as gas exchange parameters in response to Si application exhibited a quadratic behavior (150 g ha^−1^ Si, Table 1), consistent with studies on *Poaceae* species such as wheat [55] and sorghum [24].

When absorbed by the plant, Si is deposited in the epidermal cells and guard cells of stomata, forming a cuticle-Si double layer [56], which can significantly affect gas exchange, as observed in the present study (Table 1, Figure 2). Literature reports on Si effects on gas exchange parameters (*A*, *E*, and g_s_) are contradictory. Some studies indicate that Si enhances photosynthetic rate and stomatal conductance, while others report reductions in these traits depending on dose and environmental conditions [24,57,58,59]. In our study, although the highest Si dose (300 g ha^−1^) reduced *A*, *E*, and g_s_, this response did not translate into lower crop performance. Under water deficit, Si deposition in the leaf cuticle can act as a physical barrier that limits stomatal opening and transpiration, improving water-use efficiency rather than restricting carbon assimilation [57,58,59]. Furthermore, grain yield did not peak at the maximum Si level but at the intermediate dose (150 g ha^−1^), where gas exchange was still stimulated (Table 1, Figure 4c). At higher doses, the decline in *A* was counterbalanced by substantial improvements in root development (length, surface area, and volume) and by the increase in thousand-grain weight (Figure 4b). These traits enhanced water and nutrient uptake, delayed leaf senescence, and sustained assimilate translocation to kernels, thereby supporting grain filling despite lower instantaneous photosynthesis. Such contrasting responses—reduced stomatal conductance but improved root growth and assimilate partitioning—have also been described as adaptive mechanisms in maize and other *Poaceae* species [24,60,61].

These conflicting results regarding gas exchange are attributed to species variability, with a tendency for Si to reduce transpiration (*E*) in plants receiving high Si concentrations [57], thus increasing maize tolerance to water deficit [58]. Such effects may be due to Si polymerization on the leaf surface, forming a crust that reduces gas exchange; higher Si concentrations in solution increase the risk of polysyllasic acid and silica gel formation [59]. Additionally, some plants under stress develop survival mechanisms, and our results showed that stomatal conductance (g_s_) and transpiration (*E*) were efficient indicators of potential stress in maize (Figure 2a and Figure 3b). According to Amaral et al. [62], g_s_ is an important factor, as stomata are the main pathway for gas exchange between the atmosphere and the interior of plants and constitute a major channel for water loss (*E*). Moreover, g_s_ regulates gas exchange and is directly related to photosynthetic activity (*A*) [63].

In plants, the root system is essential, providing support in the soil and constituting the root–soil interface, thereby influencing water and nutrient uptake, regulation, and storage [64,65]. Furthermore, root functions directly affect crop productivity, and thus, studies of the root system are important for agriculture to develop techniques that enhance root production and translate into increased crop yield [66,67]. Roots participate in nutrient cycling and soil organism activity [68,69]. On the other hand, roots are the first organs to detect soil drying, triggering chemical and hydraulic signaling that modulates plant morphology and physiology. Consequently, root traits are mediated by factors such as seeding density, plant genetics, production system, agronomic practices, and edaphoclimatic conditions [70].

However, the study and measurement of root morphological traits are essential for quantifying plant growth and development. Root architecture variables are primarily collected under controlled conditions (i.e., greenhouse) and during early phenological stages, as manual measurement of the root system in fully developed plants can be laborious and imprecise [71,72,73]. Therefore, the minirhizotron technique gains relevance for root evaluation, enabling image acquisition of maize roots and the quantification of root length, specific surface area, and volume (Figure 2 and Figure 4), as performed in the study by Medrado et al. [74].

In the present study, the application of 300 to 600 g ha^−1^ of Si resulted in substantial increases of 61%, 108%, and 119% in root system parameters (length, specific surface area, and volume, respectively) compared to the control (no Si application), reaching maximum values of 300.62 mm (root length with 300 g ha^−1^ Si), 529.25 mm^2^ (surface area), and 11.02 mm^3^ (volume with 600 g ha^−1^ Si). These benefits may be attributed to Si ability to stimulate lateral root production through the accumulation of abscisic acid [75,76], as well as its potential to enhance root exudate production, improving soil biological properties [15]. In maize, Si application has been shown to increase root length and the number of roots per plant, helping to mitigate water deficit stress [60], as was observed in both growing seasons of this study (Figure 1(c1) and Figure 2(c2)). In line with the literature, beneficial effects of Si on root morphology have also been reported for other *Poaceae* species such as sorghum [61] and wheat [77], supporting the consistency of Si in enhancing maize root development (Figure 2 and Figure 4).

Although the highest Si dose (300 g ha^−1^) reduced gas exchange parameters (*A*, *E*, and g_s_), this effect can be explained by the formation of a cuticle-Si double layer and possible polymerization of Si on the leaf surface, which reduce transpiration and stomatal conductance as a tolerance mechanism under water deficit [57,58]. This physiological adjustment does not necessarily indicate lower plant performance; instead, it reflects a water-saving strategy that can be beneficial under the drought conditions observed in both seasons. At the same time, Si application markedly stimulated root development (length, surface area, and volume), which enhances the plant’s capacity to absorb water and nutrients from the soil, thereby compensating for the reduced gas exchange. Similar contrasting responses—reduced stomatal conductance but enhanced root growth—have been reported as adaptive mechanisms in maize and other *Poaceae* species [24,60,61]. Therefore, the observed results are not contradictory but rather indicate a coordinated strategy in which Si modulates both shoot and root traits to improve drought resilience.

The average contents of K (21.43 g kg^−1^) and Cu (21.85 mg kg^−1^) observed in this study (Table 2) were within the adequate range (13–30 g kg^−1^) for K and near the upper limit of adequacy (6–20 mg kg^−1^) for Cu, according to Prado [49]. However, the Si average content (6.90 g kg^−1^) (Table 2) was below the average levels typically reported for this crop [78].

Si uptake in maize plants at the application rate of 600 g ha^−1^ led to increases of 146% and 304% compared to the control (Figure 4a). In this regard, several studies have highlighted the importance of using stabilizers in Si-containing solutions, such as sorbitol, due to its humectant action in spray mixtures. The enhancement of the spray solution is attributed to sorbitol (used at 10% *v*/*v*), a polyol employed as a stabilizer that delays drying of the solution while lowering the deliquescence point on the leaf surface, thereby prolonging the absorption process [24,79,80] by reducing water evaporation on the leaf surface [81] and slowing Si polymerization [82,83]. Moreover, this low molecular weight sugar—sorbitol—acts in Si solubilization at the membrane level, slightly enhancing its phloem mobility [24]. Additionally, sorbitol reduces the pH, minimizing the formation of polysilicic acid [83], as it stabilizes monomeric forms of Si (H_4_SiO_4_) by acidifying the spray solution (pH 5.5 ± 0.2), which facilitates the equilibrium among the following Si species: H_2_SiO_4_^2−^ → H_3_SiO_4_^−^ → H_4_SiO_4_ [84].

The results regarding Si uptake suggest that the application of soluble sources—such as SiKCu stabilized with sorbitol, at low Si concentrations and without polymerization—may be responsible for the increased Si absorption in maize plants under field conditions, particularly in environments prone to dry spells (i.e., temporary droughts). This finding demonstrates practical implications for agriculture, expanding the potential use of this element for maize cultivation, especially given the low application rates (0.15 to 0.60 kg ha^−1^) and the absence of environmental risk [85], in contrast to insoluble sources, such as calcium silicate (>1 Mg ha^−1^), which reduce plant Si uptake and increase production costs [86,87].

It is important to highlight that Si uptake varies among plant species [85], with some species accumulating substantial Si amounts in specific foliar and root tissues [88]. Within the *Poaceae* family, the average Si content accumulated by *Zea mays* is 0.58% [89], categorizing it as an intermediate Si accumulator (0.5–1%) [88], due to its passive transport system [90]. The gene *Lsi1* encodes a bidirectional Si transporter that passively facilitates uptake driven by concentration gradients [90]. Homologs of *Lsi1* identified and characterized in *Zea mays*, similar to those in rice (*Oryza sativa*), include *ZmLsi1*, which is expressed in maize roots and depends on root type (e.g., seminal roots) [91].

The redistribution of Si within maize plants may also be attributed to the action of identified transporter genes, whereby Si transport is regulated via influx (*Lsi1*) and efflux (*Lsi2*) mechanisms ([92,93]. The *Lsi1* gene, as a bidirectional channel-type transporter, facilitates the passive movement of Si across the plasma membrane—from the external solution (apoplast) into plant cells—via concentration gradients. All known *Lsi1* transporters are rice homologs, with *ZmLsi1* being specific to maize [90,94]. Moreover, *Lsi1* belongs to the Nodulin26-like intrinsic protein III (NIP-III) subfamily of aquaporins, a class of channel-forming membrane proteins that facilitate passive transport of water and/or small uncharged solutes, such as monosilicic acid (H_4_SiO_4_) [58,95].

However, *Lsi2* is an active transporter, requiring energy expenditure [93], as H_4_SiO_4_ permeates the plasma membrane in an antiport mechanism with H^+^ ions. Thus, the transport of H_4_SiO_4_ to the endodermis via the symplastic route is mediated by the *ZmLsi2* gene [90,96,97]. Nevertheless, the complete structural and functional characteristics of *Lsi2* have yet to be fully elucidated [94]. These insights into gene functionality contribute to a better understanding of Si redistribution within maize plants. Recent studies have demonstrated increased Si content resulting from foliar application, with quadratic response patterns—consistent with the findings of the present study (Figure 4a) and also observed in sorghum [98].

The increase in maize grain yield may be attributed to the number (three) of foliar applications performed throughout the crop cycle, which likely enhanced leaf number and/or total leaf area by delivering Si directly to the canopy. This technique, as applied in this study, supports the biological effectiveness of Si, as multiple foliar applications (three to four) throughout the growing season have been reported to increase the productivity of annual crops [99]. This may explain the observed yield increase in maize (Figure 4c) following the application of 150 g ha^−1^ of Si, divided into three foliar sprays during the crop cycle.

Other effects of Si contributing to productivity enhancement may be associated with the expression of genes such as *AMT1*, *CLC1*, *GS2*, and *NRT1*, which are involved in key metabolic processes (e.g., amino acid synthesis). While most studies focus on Si’s role in mitigating abiotic and biotic stress, Si has also been shown to directly influence core physiological processes in plants [100] by regulating genes linked to primary metabolism and yet unknown functions, even under non-stressful conditions [101,102,103]. Furthermore, Si can alter gene expression associated with glycolysis, cell wall biosynthesis, and the regulation of metabolic pathways (including amino acid, nitrogen, and defense hormone metabolism) [104]. Additionally, Si may increase the affinity for macromolecular binding (proteins, lipids, and polyphenols), thus contributing to higher crop productivity [105,106].

Such positive effects on productivity have been reported in the recent literature on annual crops of the *Poaceae* family, such as sorghum [24,98] and wheat [40,107]; however, those studies were conducted under controlled greenhouse conditions. Promising results from foliar Si application in maize have also been reported by Idrees et al. [60] and Teixeira et al. [54], though under imposed drought conditions in greenhouse experiments. By contrast, the present study, conducted under natural field conditions, confirms and complements those findings, as Si application mitigated the effects of naturally occurring water deficits (Figure 1(c1,c2)) and translated into higher maize grain productivity compared to the untreated control (Figure 4c).

Based on the negative effects of water deficit—such as increased leaf senescence and abscission [108], reduced biomass production [109], cell elongation, and leaf rolling [110]—one of the initial strategies to mitigate these effects in maize involves enhancing silicon (Si) uptake by plants through the use of efficient application techniques [54]. Through Si absorption in stressed plants, improvements are observed in photochemical efficiency, relative water content, and leaf angle reduction. These changes contribute to improved plant architecture, resulting in more erect leaves that intercept more solar radiation in the canopy while reducing self-shading [18], due to increased silicification of leaf tissues [111].

These benefits associated with Si application may positively influence photosynthetic performance and energy production, which are essential for the activity of nutrient transporters, thereby stimulating greater nutrient uptake [54,112]. In addition, improved nutritional status likely enhances biomass production, as nutrients support the synthesis of compounds such as carotenoids, which play both enzymatic and structural roles in plant physiology and antioxidant defense systems [54,113,114]. Thus, the benefits of Si in improving photosynthetic capacity, biomass conversion, and nutrient translocation contribute to increased grain weight [115] (i.e., larger grain size → higher thousand-grain weight) (Figure 4b, Table 2) resulting from improved water use efficiency [116,117]. Similar increases in thousand-grain weight have also been reported in other *Poaceae* species [26,40,60].

From an economic perspective, foliar application of Si has proven to be a viable alternative, resulting in positive differential profits (DP) (Figure 4d) due to the lower required dosage [86], the importance of applying economically compensatory doses [118], and its contribution to agroecosystem sustainability [119]. However, studies on the economic feasibility of foliar application of potassium and copper silicate (SiKCu) stabilized with sorbitol in maize remain scarce. Nevertheless, positive DP outcomes have been reported in other annual monocots such as rice [119] and pearl millet [120]. This highlights the importance of assessing DP when using innovative foliar Si application strategies, thus enabling producers to make informed decisions and improve financial management of this input, ultimately enhancing foliar fertilizer efficiency [19,20,21,121].

Furthermore, Si may have contributed to more efficient water use during periods of low water availability, such as in off-season maize (second crop season), thereby enhancing grain productivity. Therefore, we accept the hypothesis that foliar application of Si enhances its absorption by the plant, modulates gas exchange, and benefits development-related parameters (e.g., root system and thousand-grain weight), resulting in yield increases and positive differential profit. These findings support future perspectives raised by Souza Júnior et al. [85] and Teixeira et al. [54], who emphasized the need for field-based research to expand the efficient use of Si in sustainable maize cultivation, particularly in regions affected by natural drought conditions.

## 4. Materials and Methods

### 4.1. Characterization of the Experimental Area

The experiments were conducted at the experimental field of the School of Agronomy at the Federal University of Goiás (EA/UFG), located in the municipality of Goiânia, state of Goiás, Brazil (16°35′ S and 49°21′ W, 730 m altitude). The regional climate is classified as Aw (tropical savanna, megathermic), characterized by dry winters (May to September) and rainy summers (October to April) [122]. Over the past 36 years, the average annual precipitation has been 1495 mm [123].

The soil in the study area was classified as a Latossolo Amarelo Distrófico, according to the Brazilian Soil Classification System (SiBCS) [124], with a sandy clay loam texture (clay: 320 g kg^−1^; silt: 120 g kg^−1^; sand: 560 g kg^−1^), corresponding to a Ferralsol in the World Reference Base for Soil Resources [125]. For soil chemical characterization, samples were collected at depths of 0.0–0.2 m and 0.20–0.40 m prior to the establishment of the experiments, for both growing seasons. The maize experiments were conducted at the same location in two consecutive harvests (2020 and 2021), and after harvest, soybeans were sown in the no-tillage system. The chemical analysis was performed following the methodology proposed by Teixeira et al. [126], and the results are presented in Table 3. The soil Si content was 2.50 mg dm^−3^ (0.00–0.20 m) and 2.43 mg dm^−3^ (0.20–0.40 m) when extracted with calcium chloride (CaCl_2_), and 2.92 mg dm^−3^ (0.00–0.20 m) and 5.09 mg dm^−3^ (0.20–0.40 m) when extracted with acetic acid (CH_3_COOH), according to the method described by Kilmer [127].

### 4.2. Climate Assessments

Daily data on the meteorological variables of rainfall (R, mm), mean air temperature (Ta, °C), maximum (TM, °C) and minimum (Tm, °C) temperatures, relative humidity (RH, %), and wind speed (u_2_, m s^−1^) were collected from the automatic Agrometeorological Station of EA/UFG, located 2.40 km from the study area. Based on these climatic variables, the maize water balance was calculated daily according to the methodology described by Thornthwaite and Mather [45], with reference evapotranspiration (ET_0_, mm day^−1^) estimated using the Penman–Monteith method proposed by Allen [128] and described in Equation (1):

(1) $${ET}_{0}=\frac{\left(0.480\;∆\;\left(R_{n}-G\right)+\gamma \;\right)\left(\frac{\;900\;u_{2}}{T_{a}\;+\;273}\;\right)\left(e_{s}-\;e_{a}\right)}{∆+\;\gamma \;(1+0.34\;u_{2})}$$

where ET_0_ is the reference evapotranspiration (mm day^−1^); ∆ is the slope of the vapor pressure curve (kPa °C^−1^); R_n_ is the net radiation balance, including incoming shortwave solar radiation and outgoing longwave terrestrial radiation (MJ m^−2^ day^−1^); G is the soil heat flux density (MJ m^−2^ day^−1^); γ is the psychrometric constant (kPa °C^−1^); u_2_ is the average wind speed measured at a 2 m height (m s^−1^); T_a_ is the mean daily air temperature (°C); e_s_ and e_a_ are the saturation vapor pressure and actual vapor pressure, respectively (kPa); and e_s_ − e_a_ is the vapor pressure deficit (kPa).

The soil water content values at field capacity (θ_cc_, m^3^ m^−3^) and permanent wilting point (θ_PWP_, m^3^ m^−3^) were calculated according to the methodology described by Arruda et al. [129], as shown in Equations (2) and (3):

(2) $$\theta_{CC}=\frac{3.1+\left(0.629\;*\;AS\right)-0.0034\;*\;\left({AS}^{2}\right)}{100}$$

(3) $$\theta_{PWP}=\frac{\left(\frac{\;398.6\;*\;AS}{1.308.1\;+\;AS}\;\right)}{100}$$

where AS is the sum of clay and silt content (%).

The available water capacity (AWC) was calculated following the method proposed by Pereira et al. [130], as shown in Equation (4):

(4) $$AWC=\left(1.000\;*\;\theta_{CC}-\;\theta_{PWP}\;\right)\;*\;Ze$$

where θ_cc_ is the soil water content at field capacity (0.24194, m^3^ m^−3^); θ_PWP_ is the soil water content at the permanent wilting point (0.12971, m^3^ m^−3^); and Ze is the effective rooting depth of maize (0.80 m), according to Camargo et al. [131].

The duration of the maize phenological stages followed the classification provided by [11], considering a four-month crop cycle. Crop evapotranspiration (ETc, mm day^−1^) was calculated as the product of reference evapotranspiration (ET_0_) and the crop coefficient (Kc). Different Kc values were used for each developmental stage of maize, adopting 0.40 for crop establishment (Stage I); 0.85 during vegetative growth (Stage II); 1.20 during flowering (Stage III); 0.85 during grain filling (Stage IV); and 0.60 during ripening and senescence (Stage V) [11].

The Ze was considered based on the maximum root depth (0.80 m) observed during the maize flowering stage (Stage III) [131]. Relative soil water storage (STO/TAW) expresses the fraction of available water between field capacity (1.0) and permanent wilting point (0.0). This index is calculated by the ratio between the soil water storage (STO) and the total available water capacity (TAW), as described by Xavier et al. [20,46].

### 4.3. Experimental Project

The experimental design followed a randomized block arrangement in a 2 × 5 factorial scheme with four replications. The first factor corresponded to the growing seasons 2020 and 2021, and the second factor consisted of foliar fertilization treatments containing silicon (0—control, 150, 300, 450, and 600 g ha^−1^ of Si), applied in the form of potassium and copper silicate (SiKCu: Si = 107 g L^−1^; K_2_O = 34.7 g L^−1^; Cu = 14.9 g L^−1^; pH = 11.8), stabilized with sorbitol (C_6_H_14_O_6_), at a concentration of 10% *v*/*v*.

The Si doses used in this study were established based on prior work by Flores et al. [19], using SiKCu also stabilized with sorbitol. In that study, the authors reported no polymerization effects in the spray solution applied to crops. For each Si treatment, the concentrations of potassium (K) and copper (Cu) were balanced using potassium chloride and copper oxide, respectively, to isolate the effect of silicon concentration. The Si spray solution was prepared without pH adjustment to simulate practical field conditions and improve operational feasibility. This approach aimed to assess the efficiency of silicon stabilization in solution in the presence of the polyol (sorbitol).

Each experimental unit consisted of twelve rows, each 5 m long, with an inter-row spacing of 0.45 m. Silicon foliar applications were split into three phenological stages: the first at the four fully expanded leaf stage (V4); the second at the eight-leaf stage (V8); and the final application at the onset of the reproductive phase (R1) [132]. Foliar applications were performed immediately after spray solution preparation using a CO_2_-pressurized backpack sprayer [equipped with a 3 m wide spray boom and six flat-fan nozzles (XR 110.02) spaced at 0.5 m], delivering a spray volume of 150 L ha^−1^. All sprays were directed to the adaxial side of the leaves to simulate mechanized spraying. Applications were conducted between 8 and 10 a.m., under temperature conditions ranging from 20 to 23 °C and relative humidity >85%, which are considered favorable for foliar spraying [49].

The maize hybrid used (B2433 PWU) is classified as ultra-early, recommended for medium- to high-input systems, with high yield stability, suitable for the first (summer) and second seasons, and indicated for both grain and silage production [133,134]. Sowing was carried out in the first half of March for both growing seasons (2020 and 2021), and harvesting took place in the second half of June. Seed germination potential was assessed using the tetrazolium test [135], resulting in 100% viability and 95% vigor for both seasons. Planting was performed at a rate of 2.8 viable seeds per linear meter, with a row spacing of 0.45 m, targeting a final stand of 55,000 plants ha^−1^, as recommended for the crop.

Based on the results of chemical and particle-size soil analysis, lime and mineral fertilization requirements were determined following the recommendations of Sousa and Lobato [136] for maize production. At sowing, fertilization was performed with the application of 30 kg ha^−1^ of N, 120 kg ha^−1^ of P_2_O_5_, and 60 kg ha^−1^ of K_2_O in the seed furrow, using urea, monoammonium phosphate (MAP), and potassium chloride (KCl), respectively. Topdressing fertilization consisted of nitrogen (N) and potassium (K_2_O) at rates of 180 and 90 kg ha^−1^, respectively, applied in two equal splits: 50% between V4 and V6 stages, and the remaining 50% between V8 and V10 stages.

### 4.4. Evaluations

After plant emergence, acrylic tubes approximately 0.60 m in length and 70 mm in diameter were installed in each experimental unit. The 0.40 m portion of the tube inserted into the soil was used for minirhizotron-based root imaging to evaluate root development at the end of the crop cycle in both growing seasons. The acrylic tube was installed with its upper edge approximately 0.05 m above the soil surface and was sealed with a cap at both ends to prevent the entry of debris. This setup aimed to block light during image acquisition and to standardize the scanner reading height, using the soil surface line as the image reference point rather than the tube upper edge.

To improve image precision and depth classification, a 0.20 m reference rod was used. Root imaging using the minirhizotron method was conducted with the CI-600 CanoScan root scanner (CID Bio-Science, Version 3.1.19, Camas, WA, USA), following the methodology described by Medrado et al. [74]. The scanner was connected to a laptop for in-field storage of the collected image database. Subsequently, the images were analyzed using RootSnap software, which enables manual tracing of root length (cm), specific surface area (mm^2^), and volume (mm^3^) within the effective depth range of 0–0.40 m.

Seven days after the last silicon (Si) application, at the R1 phenological stage of maize, and in each growing season, 30 diagnostic leaves (middle third of the fully expanded leaf below the ear) were collected per experimental unit to assess the nutritional status of K, Cu, and Si, following the methodology described by Prado [49]. Leaf samples were washed with running water, followed by distilled water to remove surface impurities. Subsequently, the samples were oven-dried in a forced-air circulation oven at 65 °C for 72 h and then ground in a Wiley-type mill using a 2 mm mesh screen for chemical analysis. Potassium and copper concentrations were determined according to the protocol proposed by Silva [137], and silicon content was analyzed following the method described by Kraska and Breitenbeck [138].

At the same phenological stage mentioned previously (R1), physiological evaluations of the plants were performed by measuring gas exchange parameters: stomatal conductance (g_s_, mol H_2_O m^−2^ s^−1^), transpiration rate (*E*, mmol H_2_O m^−2^ s^−1^), and net photosynthetic rate (*A*, μmol CO_2_ m^−2^ s^−1^). Measurements were taken using an infrared gas analyzer (IRGA), model LCpro-SD/iFL Portable, ADC Bioscience LTDA, Hodderdon, UK, equipped with a 6.25 cm^2^ leaf chamber. In each experimental unit, readings were conducted between 8:00 and 10:00 a.m. on three diagnostic leaves in good phytosanitary condition, as recommended by Flores et al. [24]. The photosynthetic photon flux density was fixed at 2000 µmol m^−2^ s^−1^.

At harvest, the thousand-grain weight (TGW) and grain yield were evaluated after the crop reached physiological maturity, according to the methodology of Lima [132]. Harvesting was carried out by collecting all ears from a 10 m linear section of each experimental unit. The ears were threshed, and the grains were dried and weighed. The data were then corrected to 13% moisture content on a wet basis and converted to kg ha^−1^.

### 4.5. Economic Analysis

The economic analysis was conducted using the partial budgeting method, as described by Noronha [139]. This method accounts for the effects of additional costs and revenues relative to a baseline, providing differential profits (DP) as the economic indicator, according to Equation (5):

(5) $$DP=\left(Rd-\;Cd\;\right)$$

where Rd = differential revenue, calculated for each treatment as the product of the yield differential obtained in the treatment relative to the control and the deflated historical average price per kilogram of maize. The prices correspond to the monthly average reference prices for Brazil from 2015 to 2025 [140], deflated by the price evolution indicator according to the General Price Index–Internal Availability (IGP-DI) [141].

Cd = differential cost, obtained based on the prices of inputs used in each treatment according to their respective concentrations, relative to the control. These prices were directly obtained from the research budgets. The input cost included the operational cost of spraying [142], updated to 2025, and corresponding to the three spray applications performed.

All values were converted to US dollars at the exchange rate of 1 April 2025, where USD 1.00 = BRL 5.71. Thus, the differential profit (DP) was obtained from Equation (5).

### 4.6. Statistical Analysis

The data were subjected to analysis of variance (ANOVA) using the F-test at a 5% significance level, performed with the AgroEstat statistical package [143]. When statistically significant differences were detected among treatments, means of qualitative parameters were compared using Tukey’s test at 1% and 5% probability levels. For quantitative parameters showing significance, polynomial regression analysis was conducted.

## 5. Conclusions

Foliar fertilization with silicon at a dose of 150 g ha^−1^ increases transpiration rate by up to 9%, net photosynthetic rate by 13%, and grain yield of maize by 10% after two growing seasons, regardless of the water deficit experienced during the crop cycle. At this dose, silicon application is economically viable, yielding the highest differential profit (USD 97.11 ha^−1^).

In conclusion, foliar fertilization with silicon is an agronomically and economically viable strategy for efficient maize grain production during the second growing season in tropical regions.

## Figures and Tables

**Table 1:** Net photosynthesis rate (*A*), transpiration (*E*), stomatal conductance (g_s_), root length, and specific root surface area and root volume of maize, as a function of growing season and foliar application of silicon.

Treatments	*A*	*E*	g_s_	Length	Area	Volume
Growing Season (GS)	µmol CO_2_ m^−2^ s^−1^	mmol H_2_O m^−2^ s^−1^	mol H_2_O m^−2^ s^−1^	mm	mm^2^	mm^3^
2020	59.94 ± 2.10 a	6.84 ± 0.42 a	0.38 ± 0.03 a	254.45 ± 12.43 a	387.75 ± 6.64 a	7.25 ± 0.34 a
2021	53.90 ± 2.40 b	6.31 ± 0.39 a	0.31 ± 0.02 b	249.55 ± 15.64 a	367.15 ± 11.02 a	7.05 ± 0.33 a
F	13.76 **	3.19 ^ns^	18.36 **	0.09 ^ns^	0.88 ^ns^	0.14 ^ns^
Si dose, g ha^−1^ (Si)						
0	73.36 ± 1.83 b	7.77 ± 0.42 ab	0.45 ± 0.01 b	187.25 ± 8.41 d	254.25 ± 10.44 e	5.04 ± 0.27 c
150	82.72 ± 2.51 a	8.46 ± 0.06 a	0.54 ± 0.03 a	200.25 ± 6.78 c	277.62 ± 5.15 d	5.36 ± 0.16 c
300	53.82 ± 2.12 c	6.53 ± 0.52 bc	0.36 ± 0.03 c	300.62 ± 22.42 a	374.50 ± 12.53 c	5.32 ± 0.21 c
450	36.35 ± 2.93 d	4.63 ± 0.54 d	0.19 ± 0.01 d	287.75 ± 28.58 a	446.62 ± 8.35 b	9.00 ± 0.43 b
600	38.33 ± 1.84 d	5.46 ± 0.47 cd	0.18 ± 0.02 d	284.12 ± 3.99 b	529.25 ± 7.07 a	11.02 ± 0.59 a
F	129.49 **	22.73 **	81.10 **	8.41 **	26.85 **	20.45 **
Interaction effects (GS × Si)						
F	37.98 **	10.12 **	27.70 **	0.24 ^ns^	0.20 ^ns^	0.08 ^ns^
Average	59.92	6.57	0.34	252.00	376.45	7.15
C.V.	9.04	14.30	14.57	20.79	16.69	23.72

^ns^ and **—not significant at 5% probability by the Tukey test and significant at 1% probability by the F test, respectively. Mean standard error (±). C.V.—coefficient of variation. Different letters in the column differ from each other.

**Table 2:** Foliar contents of silicon (Si), 1000 grain weight (1000 G) and grain yield of maize, as a function of the growing season and foliar application of silicon.

Treatments	Si	1000 G	Yield
Growing Season (GS)	g kg^−1^	g	kg ha^−1^
2020	6.44 ± 0.50 b	191.80 ± 3.79 a	5544 ± 65.32 a
2021	7.36 ± 0.45 a	193.86 ± 2.85 a	5523 ± 62.98 a
F	5.14 *	0.54 ^ns^	2.33 ^ns^
Si dose, g ha^−1^ (Si)			
0	3.85 ± 0.70 c	176.43 ± 1.91 c	5382 ± 28.96 c
150	6.58 ± 0.20 b	186.42 ± 3.59 bc	5938 ± 81.40 a
300	5.90 ± 0.35 b	192.50 ± 3.09 b	5570 ± 57.73 b
450	6.78 ± 0.45 b	209.55 ± 5.67 a	5424 ± 102.88 bc
600	11.40 ± 0.75 a	199.26 ± 2.34 ab	5353 ± 49.78 c
F	36.37 **	16.14 **	18.69 *
Interaction effects (GS × Si)			
F	9.43 **	1.68 ^ns^	8.19 **
Average	6.90	192.83	5545
C.V.	18.83	4.58	2.68

^ns^, ** and *—not significant at 5% probability by the Tukey test and significant at 1 and 5% probability by the F test, respectively. Mean standard error (±). C.V.—coefficient of variation. Different letters in the column differ from each other.

**Table 3:** Soil chemical attributes before the installation of the experiments in the 2020 and 2021 growing seasons.

***2020 Growing Season***									
Layer	Clay	Sand	Silt	Cu	Fe^2+^	Mn	Zn	OM	pH
m	g kg^−1^	g kg^−1^	g kg^−1^	mg dm^−3^	mg dm^−3^	mg dm^−3^	mg dm^−3^	g kg^−1^	(CaCl_2_)
0.00–0.20	320.00	560.00	120.00	2.00	53.00	58.00	4.00	18.00	4.80
0.20–0.40	440.00	470.00	90.00	2.00	44.00	46.00	1.80	21.00	4.80
Layer	P	K	Ca^2+^	Mg^2+^	H+Al	Al^3+^	CEC	m	BS
m	mg dm^−3^	mg dm^−3^	cmol_c_ dm^−3^	cmol_c_ dm^−3^	cmol_c_ dm^−3^	cmol_c_ dm^−3^	cmol_c_ dm^−3^	%	%
0.00–0.20	15.20	160.00	2.40	1.10	4.30	0.20	8.20	4.90	47.60
0.20–0.40	3.40	110.00	2.00	1.00	4.30	0.10	7.60	3.00	43.30
***2021 Growing Season***									
Layer	Clay	Sand	Silt	Cu	Fe^2+^	Mn	Zn	OM	pH
m	g kg^−1^	g kg^−1^	g kg^−1^	mg dm^−3^	mg dm^−3^	mg dm^−3^	mg dm^−3^	g kg^−1^	(CaCl_2_)
0.00–0.20	320.00	560.00	120.00	1.80	27.00	29.00	3.30	29.00	5.10
0.20–0.40	440.00	470.00	90.00	1.80	30.00	31.00	4.00	18.00	4.90
Layer	P	K	Ca^2+^	Mg^2+^	H+Al	Al^3+^	CEC	m	BS
m	mg dm^−3^	mg dm^−3^	cmol_c_ dm^−3^	cmol_c_ dm^−3^	cmol_c_ dm^−3^	cmol_c_ dm^−3^	cmol_c_ dm^−3^	%	%
0.00–0.20	25.10	115.00	2.70	1.50	2.80	0.00	7.30	0.00	61.80
0.20–0.40	8.30	64.00	1.80	1.20	3.10	0.10	6.30	3.00	50.70

Cu = copper; Fe = iron; Mn = manganese; Zn = zinc; OM = organic matter of soil; P = phosphorus (Mehlich); K = potassium; Ca = calcium; Mg = magnesium; H = hydrogen; Al = aluminum; CEC = cation exchange capacity; m = saturation by Al; BS = base saturation.

## Data Availability

The original contributions presented in this study are included in the article. Further inquiries can be directed to the corresponding author.

## Abbreviations

The following abbreviations are used in this manuscript:

A	Photosynthetic rate
ANOVA	Analysis of variance
AS	Sum of clay and silt content
Aw	Tropical savanna, megathermic
Cd	Differential cost
DP	Differential profits
EA/UFG	School of Agronomy at the Federal University of Goiás
E	Transpiration rate
ETc	Crop evapotranspiration
ET_0_	Reference evapotranspiration
es	Saturation vapor pressure
ea	Actual vapor pressure
γ	Psychrometric constant
G	Soil heat flux density
gs	Stomatal conductance
IGP-DI	General Price Index—Internal Availability
Kc	Crop coefficient
KCl	Potassium chloride
MAP	Monoammonium phosphate
R	Rainfall
R1	Onset of the reproductive phase
Rd	Differential revenue
RH	Relative humidity
Rn	Net radiation balance
SiBCS	Brazilian Soil Classification System
SiKCu	Potassium and copper silicate
STO/TAW	Relative water storage in soil
STICS	Multidisciplinary Simulator for Standard Cultures
Ta	Average temperature
TGW	Thousand-grain weight
TM	Maximum temperature
Tm	Minimum temperature
u_2_	Wind speed
UV	Ultraviolet radiation
V4	First at the four fully expanded leaf stage
V8	Second at the eight-leaf stage
Ze	Effective rooting depth
θ_cc_	Field capacity
θ_PWP_	Permanent wilting point
∆	Slope of the vapor pressure curve

## References

[1] Bokor B., Santos C.S., Kostoláni D., Machado J., Silva M.N., Carvalho S.M.P., Vaculík M., Vasconcelos M.W. (2021). Mitigation of Climate Change and Environmental Hazards in Plants: Potential Role of the Beneficial Metalloid Silicon. J. Hazard. Mater..

[2] Pugnaire F.I., Morillo J.A., Peñuelas J., Reich P.B., Bardgett R.D., Gaxiola A., Wardle D.A., van der Putten W.H. (2019). Climate Change Effects on Plant-Soil Feedbacks and Consequences for Biodiversity and Functioning of Terrestrial Ecosystems. Sci. Adv..

[3] Blöschl G., Hall J., Viglione A., Perdigão R.A.P., Parajka J., Merz B., Lun D., Arheimer B., Aronica G.T., Bilibashi A. (2019). Changing Climate Both Increases and Decreases European River Floods. Nature.

[4] Le Gouis J., Oury F., Charmet G. (2020). How Changes in Climate and Agricultural Practices Influenced Wheat Production in Western Europe. J. Cereal Sci..

[5] Cottrell R.S., Nash K.L., Halpern B.S., Remenyi T.A., Corney S.P., Fleming A., Fulton E.A., Hornborg S., Johne A., Watson R.A. (2019). Food Production Shocks across Land and Sea. Nat. Sustain..

[6] Costa M.G., Prado R.M., Sarah M.M.S., Souza A.E.S., Souza Júnior J.P. (2023). Silicon Mitigates K Deficiency in Maize by Modifying C, N, and P Stoichiometry and Nutritional Efficiency. Sci. Rep..

[7] Bassu S., Brisson N., Durand J., Boote K., Lizaso J., Jones J.W., Rosenzweig C., Ruane A.C., Adam M., Baron C. (2014). How Do Various Maize Crop Models Vary in Their Responses to Climate Change Factors?. Glob. Change Biol..

[8] Silva P.P.G., Andrade C.L.T., Magalhães B.G., Gontijo Neto M.M., Melo B.F. (2016). Produtividade Potencial e Variabilidade da Produtividade de Milho, em Regime de Sequeiro, em Rio Verde, Goiás.

[9] Erenstein O., Jaleta M., Sonder K., Mottaleb K., Prasanna B.M. (2022). Global Maize Production, Consumption and Trade: Trends and R&D Implications. Food Secur..

[10] FAO Crops and Livestock Products: Maize. https://www.fao.org/faostat/en/#data/QCL/visualize.

[11] FAO Maize: Water Supply and Crop Yield. https://www.fao.org/land-water/databases-and-software/crop-information/maize/en/.

[12] Conab (2025). Acompanhamento da Safra Brasileira de Grãos.

[13] Battisti R., Ferreira M.D.P., Tavares E.B., Knapp F.M., Bender F.D., Casaroli D., Alves Júnior J. (2020). Rules for Grown Soybean-Maize Cropping System in Midwestern Brazil: Food Production and Economic Profits. Agric. Syst..

[14] Lima M.A., Castro V.F., Vidal J.B., Enéas-Filho J. (2011). Aplicação de silício em milho e feijão-de-corda sob estresse salino. Rev. Ciênc. Agron..

[15] Shamshiripour M., Motesharezadeh B., Rahmani H.A., Alikhani H.A., Etesami H. (2022). Optimal Concentrations of Silicon Enhance the Growth of Soybean (*Glycine max* L.) Cultivars by Improving Nodulation, Root System Architecture, and Soil Biological Properties. Silicon.

[16] Epstein E. (2009). Silicon: Its Manifold Roles in Plants. Ann. Appl. Biol..

[17] Thakral V., Bhat J.A., Kumar N., Myaka B., Sudhakaran S., Patil G., Sonah H., Shivaraj S.M., Deshmukh R. (2021). Role of Silicon under Contrasting Biotic and Abiotic Stress Conditions Provides Benefits for Climate Smart Cropping. Environ. Exp. Bot..

[18] Ma J.F., Yamaji N. (2006). Silicon Uptake and Accumulation in Higher Plants. Trends Plant Sci..

[19] Flores R.A., Lima F.S.R., Xavier M.F.N., Bueno A.M., Andrade A.F., Souza Júnior J.P., Campos C.N.S., Cunha Júnior L.C., Abdala K.O., Prado R.M. (2024). Soluble Silicon Source via Foliar Application Improve Plant Physiology and Fruit Quality of *Solanum lycopersicum* L. *Silicon*
**2024**, *16*, 1943–1954. Silicon.

[20] Xavier M.F.N., Flores R.A., Carmo R.T., Lima M.L., Sousa R.G., Dapper F.P., Abdala K.O., Casaroli D., Momesso L., Santos G.G. (2025). Influence of Nitrogen Sources and Foliar Silicon Fertilization on Agronomic Characteristics and Differential Profit from Sugarcane Stalk Production. J. Plant Nutr..

[21] Xavier M.F.N., Flores R.A., Cruz D.R.C., Ferreira I.V.L., Castro J.P.V., Silva M.L., Muniz M.P., Silva V.B., Milagres V.A.C., Abdala K.O. (2025). Foliar Fertilization with a Soluble Silicon Source Can Alter Pigment Production in Leaves and Increase Fruit Production in Cucumbers (*Cucumis sativus* L.). J. Plant Nutr..

[22] Bityutskii N.P., Yakkonen K.L., Petrova A.I., Lukina K.A., Shavarda A.L. (2018). Silicon Ameliorates Iron Deficiency of Cucumber in a pH-Dependent Manner. J. Plant Physiol..

[23] Araújo V.S., Sousa T.K.R., Nobre R.S., Santos C.M., Negreiros K.K.S., Carvalho A.C.C., Veloso F.S., Veloso R.C., Rezende J.S. (2022). Influence of Foliar Application of Silicon on the Development and Productivity of Corn under Water Deficit in the Semi-Arid Region of Piauí. Res. Soc. Dev..

[24] Flores R.A., Arruda E.M., Damin V., Souza Júnior J.P., Maranhão D.D.C., Correia M.A.R., Prado R.M. (2018). Physiological Quality and Dry Mass Production of Sorghum Bicolor Following Silicon (Si) Foliar Application. Aust. J. Crop Sci..

[25] Freitas L.B., Coelho E.M., Maia S.C.M., Silva T.R.B. (2011). Adubação foliar com silício na cultura do milho. Rev. Ceres.

[26] Miranda P.S., Moraes T.R., Santos J.R.E., Carvalho F.D., Viana J.P., Pérez-Maluf R. (2018). Aplicação de silício na cultura do milho. Rev. Ciências Agro-Ambient..

[27] Munaro M.F., Simonetti A.P.M.M. (2016). Aplicação foliar de silício no milho 2a safra: Influência na produtividade. Rev. Cultiv. Saber.

[28] Hawerroth C., Araujo L., Bermúdez-Cardona M.B., Silveira P.R., Wordell Filho J.A., Rodrigues F.A. (2019). Silicon-Mediated Maize Resistance to Macrospora Leaf Spot. Trop. Plant Pathol..

[29] Mochko A.C.R., Silva B.N., Oliveira L.M., Silva L.C., Rodrigues F.A. (2024). Silicon-Mediated Resistance in Maize against Infection by Colletotrichum Graminicola. Plant Soil.

[30] Sarah M.M.S., Prado R.M., Teixeira G.C.M., Souza Júnior J.P., Medeiros R.L.S., Barreto R.F. (2022). Silicon Supplied Via Roots or Leaves Relieves Potassium Deficiency in Maize Plants. Silicon.

[31] Hosseini S.A., Rad S.N., Ali N., Yvin J. (2019). The Ameliorative Effect of Silicon on Maize Plants Grown in Mg-Deficient Conditions. Int. J. Mol. Sci..

[32] Oliveira K.S., Prado R.M., Guedes V.H.F. (2020). Leaf Spraying of Manganese with Silicon Addition Is Agronomically Viable for Corn and Sorghum Plants. J. Soil Sci. Plant Nutr..

[33] Campos C.N.S., Prado R.M., Roque C.G., Lima Neto A.J., Marques L.J.P., Chaves A.P., Cruz C.A. (2015). Use of Silicon in Mitigating Ammonium Toxicity in Maize Plants. Am. J. Plant Sci..

[34] Dresler S., Wójcik M., Bednarek W., Hanaka A., Tukiendorf A. (2015). The Effect of Silicon on Maize Growth under Cadmium Stress. Russ. J. Plant Physiol..

[35] Kaya C., Tuna A.L., Sonmez O., Ince F., Higgs D. (2009). Mitigation Effects of Silicon on Maize Plants Grown at High Zinc. J. Plant Nutr..

[36] Delavar K., Ghanati F., Behmanesh M., Zare-Maivan H. (2018). Physiological Parameters of Silicon-Treated Maize Under Salt Stress Conditions. Silicon.

[37] Malčovská S.M., Dučaiová Z., Bačkor M. (2014). Impact of Silicon on Maize Seedlings Exposed to Short-Term UV-B Irradiation. Biologia.

[38] Carvalho J.S., Frazão J.J., Prado R.M., Souza Júnior J.P., Costa M.G. (2022). Silicon Modifies C:N:P Stoichiometry and Improves the Physiological Efficiency and Dry Matter Mass Production of Sorghum Grown under Nutritional Sufficiency. Sci. Rep..

[39] Neu S., Schaller J., Dudel E.G. (2017). Silicon Availability Modifies Nutrient Use Efficiency and Content, C:N:P Stoichiometry, and Productivity of Winter Wheat (*Triticum aestivum* L.). Sci. Rep..

[40] Qamar R., Anjum I., Atique-ur-Rehman, Safdar M.E., Javeed H.M.R., Rehman A., Ramzan Y. (2020). Mitigating Water Stress on Wheat through Foliar Application of Silicon. Asian J. Agric. Biol..

[41] Bellido L.L. (1991). Cultivos Herbaceos.

[42] Cruz J.C., Pereira Filho I.A., Alvarenga R.C., Gontijo Neto M.M., Viana J.H.M., Oliveira M.F., Santana D.P. (2006). Manejo da Cultura do Milho.

[43] Fancelli A.L. (2015). Cultivo Racional e Sustentável Requer Maior Conhecimento Sobre Planta Do Milho. Visão Agrícola.

[44] Souza G.M., Barbosa A.M. (2015). Fatores de Estresse No Milho São Diversos e Exigem Monitoramento Constante. Visão Agrícola.

[45] Thornthwaite C.W., Mather J.R. (1955). The Water Balance.

[46] Xavier M.F.N., Flores R.A., Casaroli D., Capuchinho F.F., Dapper F.P., Carmo R.T., Lima M.L., Campos C.N.S., Santos G.G., Damin V. (2024). CO_2_ Emission and Physiological Aspects of Sugarcane Ratoon as Interactive Functions of Nitrogen and Silicon Applications. J. Plant Nutr..

[47] Birchall J.D. (1995). The Essentiality of Silicon in Biology. Chem. Soc. Rev..

[48] Souza Júnior J.P., Prado R.M., Campos C.N.S., Oliveira D.F., Cazetta J.O., Detoni J.A. (2022). Silicon Foliar Spraying in the Reproductive Stage of Cotton Plays an Equivalent Role to Boron in Increasing Yield, and Combined Boron-Silicon Application, without Polymerization, Increases Fiber Quality. Ind. Crops Prod..

[49] Prado R.M. (2021). Mineral Nutrition of Tropical Plants.

[50] Wien H.C. (2020). Abiotic Stress Effects on Vegetable Crops. The Physiology of Vegetable Crops.

[51] Parveen A., Liu W., Hussain S., Asghar J., Perveen S., Xiong Y. (2019). Silicon Priming Regulates Morpho-Physiological Growth and Oxidative Metabolism in Maize under Drought Stress. Plants.

[52] Santos A.F.B., Teixeira G.C.M., Campos C.N.S., Baio F.H.R., Prado R.M., Teodoro L.P.R., Vilela R.G., Paiva Neto V.B., Teodoro P.E. (2020). Silicon Increases Chlorophyll and Photosynthesis and Improves Height and NDVI of Cotton (*Gossypium hirsutum* L. r. Latifolium hutch). Res. Soc. Dev..

[53] Souza Júnior J.P., Prado R.M., Sarah M.M.S., Felisberto G. (2019). Silicon Mitigates Boron Deficiency and Toxicity in Cotton Cultivated in Nutrient Solution. J. Plant Nutr. Soil Sci..

[54] Teixeira G.C.M., Prado R.M., Oliveira L.T., Souza J.V.C., Rocha A.M.S. (2022). Silicon Fertigation with Appropriate Source Reduces Water Requirement of Maize under Water Deficit. Plant Soil.

[55] Ahmed M., Qadeer U., Fayayz-ul-Hassan, Fahad S., Naseem W., Duangpan S., Ahmad S. (2020). Abiotic Stress Tolerance in Wheat and the Role of Silicon: An Experimental Evidence. Agronomic Crops: Volume 3: Stress Responses and Tolerance.

[56] Keller C., Rizwan M., Davidian J., Pokrovsky O.S., Bovet N., Chaurand P., Meunier J. (2015). Effect of Silicon on Wheat Seedlings (*Triticum turgidum* L.) Grown in Hydroponics and Exposed to 0 to 30 µM Cu. Planta.

[57] Chen D., Wang S., Yin L., Deng X. (2018). How Does Silicon Mediate Plant Water Uptake and Loss Under Water Deficiency?. Front. Plant Sci..

[58] Ma J.F., Yamaji N. (2015). A Cooperative System of Silicon Transport in Plants. Trends Plant Sci..

[59] Haynes R.J. (2019). What Effect Does Liming Have on Silicon Availability in Agricultural Soils?. Geoderma.

[60] Idrees K., Aziz A., Naeem M., Azhar M.F., Farooq S., Hussain M. (2024). Combined Application of Zinc and Silicon Improved Growth, Gas Exchange Traits, and Productivity of Maize (*Zea mays* L.) Under Water Stress. Silicon.

[61] Yin L., Wang S., Liu P., Wang W., Cao D., Deng X., Zhang S. (2014). Silicon-Mediated Changes in Polyamine and 1-Aminocyclopropane-1-Carboxylic Acid Are Involved in Silicon-Induced Drought Resistance in *Sorghum bicolor* L.. Plant Physiol. Biochem..

[62] Amaral J.A.T., Rena A.B., Amaral J.F.T.D. (2006). Crescimento vegetativo sazonal do cafeeiro e sua relação com fotoperíodo, frutificação, resistência estomática e fotossíntese. Pesq. Agropec. Bras..

[63] Ferreira S.M. (2008). O Efeito do Silício na Cultura do Algodoeiro (*Gossypium hirsutum* L.): Aspectos Bioquímicos, Qualidade de Fibra e Produtividade. Ph.D. Thesis.

[64] Ober E.S., Alahmad S., Cockram J., Forestan C., Hickey L.T., Kant J., Maccaferri M., Marr E., Milner M., Pinto F. (2021). Wheat Root Systems as a Breeding Target for Climate Resilience. Theor. Appl. Genet..

[65] Zhao J., Bodner G., Rewald B., Leitner D., Nagel K.A., Nakhforoosh A. (2017). Root Architecture Simulation Improves the Inference from Seedling Root Phenotyping towards Mature Root Systems. J. Exp. Bot..

[66] Freschet G.T., Pagès L., Iversen C.M., Comas L.H., Rewald B., Roumet C., Klimešová J., Zadworny M., Poorter H., Postma J.A. (2021). A Starting Guide to Root Ecology: Strengthening Ecological Concepts and Standardising Root Classification, Sampling, Processing and Trait Measurements. New Phytol..

[67] Paez-Garcia A., Motes C.M., Scheible W., Chen R., Blancaflor E.B., Monteros M.J. (2015). Root Traits and Phenotyping Strategies for Plant Improvement. Plants.

[68] Bardgett R.D., Mommer L., De Vries F.T. (2014). Going Underground: Root Traits as Drivers of Ecosystem Processes. Trends Ecol. Evol..

[69] Faucon M., Houben D., Lambers H. (2017). Plant Functional Traits: Soil and Ecosystem Services. Trends Plant Sci..

[70] Meister R., Rajani M.S., Ruzicka D., Schachtman D.P. (2014). Challenges of Modifying Root Traits in Crops for Agriculture. Trends Plant Sci..

[71] Costa A.R. (2001). As Relações Hídricas Das Plantas Vasculares.

[72] French A., Ubeda-Tomás S., Holman T.J., Bennett M.J., Pridmore T. (2009). High-Throughput Quantification of Root Growth Using a Novel Image-Analysis Tool. Plant Physiol..

[73] Lobet G., Pagès L., Draye X. (2011). A Novel Image-Analysis Toolbox Enabling Quantitative Analysis of Root System Architecture. Plant Physiol..

[74] Medrado L.C., Santos G.G., Correchel V., Silva G.C., Flores R.A., Severiano E.C., Mesquita M., Figueiredo C.C. (2023). Evaluation of Sugarcane Root Growth Through Images Obtained via the Minirhizotron Method in a Ferralsol in the Midwest Region of Brazil. Sugar Tech.

[75] Dakora F.D., Nelwamondo A. (2003). Silicon Nutrition Promotes Root Growth and Tissue Mechanical Strength in Symbiotic Cowpea. Funct. Plant Biol..

[76] Signora L., De Smet I., Foyer C.H., Zhang H. (2001). ABA Plays a Central Role in Mediating the Regulatory Effects of Nitrate on Root Branching in Arabidopsis. Plant J..

[77] Ashfaq W., Brodie G., Fuentes S., Pang A., Gupta D. (2024). Silicon Improves Root System and Canopy Physiology in Wheat under Drought Stress. Plant Soil.

[78] Guntzer F., Keller C., Meunier J. (2012). Benefits of Plant Silicon for Crops: A Review. Agron. Sustain. Dev..

[79] Kubicki J.D., Heaney P.J. (2003). Molecular Orbital Modeling of Aqueous Organosilicon Complexes: Implications for Silica Biomineralization. Geochim. Cosmochim. Acta.

[80] Will S., Eichert T., Fernández V., Möhring J., Müller T., Römheld V. (2011). Absorption and Mobility of Foliar-Applied Boron in Soybean as Affected by Plant Boron Status and Application as a Polyol Complex. Plant Soil.

[81] D’Souza A.A., Shegokar R. (2016). Polyethylene Glycol (PEG): A Versatile Polymer for Pharmaceutical Applications. Expert. Opin. Drug Deliv..

[82] Fernández V., Brown P.H. (2013). From Plant Surface to Plant Metabolism: The Uncertain Fate of Foliar-Applied Nutrients. Front. Plant Sci..

[83] Flores R.A., Xavier M.F.N. (2023). Innovative Sources and Ways of Applying Silicon to Plants. Benefits of Silicon in the Nutrition of Plants.

[84] Kudryavtsev P.G., Figovsky O.L. (2016). Nanocomposite Organomineral Hybrid Materials. Part 2. Nanotehnol. Stroit..

[85] Souza Júnior J.P., Prado R.M., Diniz J.F., Guedes V.H.F., Silva J.L.F., Roque C.G., Alvarez R.C.F. (2022). Foliar Application of Innovative Sources of Silicon in Soybean, Cotton, and Maize. J. Soil Sci. Plant Nutr..

[86] Flores R.A., Arruda E.M., Souza Júnior J.P., Prado R.M., Santos A.C.A., Aragão A.S., Pedreira N.G., Costa C.F. (2019). Nutrition and Production of Helianthus Annuus in a Function of Application of Leaf Silicon. J. Plant Nutr..

[87] Keeping M.G. (2017). Uptake of Silicon by Sugarcane from Applied Sources May Not Reflect Plant-Available Soil Silicon and Total Silicon Content of Sources. Front. Plant Sci..

[88] Ma J.F., Takahashi E. (2002). Soil, Fertilizer, and Plant Silicon Research in Japan.

[89] Deshmukh R., Sonah H., Bélanger R.R. (2020). New Evidence Defining the Evolutionary Path of Aquaporins Regulating Silicon Uptake in Land Plants. J. Exp. Bot..

[90] Mitani-Ueno N., Ma J.F. (2021). Linking Transport System of Silicon with Its Accumulation in Different Plant Species. Soil Sci. Plant Nutr..

[91] Mitani N., Yamaji N., Ma J.F. (2009). Identification of Maize Silicon Influx Transporters. Plant Cell Physiol..

[92] Ma J.F., Yamaji N., Mitani-Ueno N. (2011). Transport of Silicon from Roots to Panicles in Plants. Proc. Jpn. Acad. Ser. B.

[93] Mitani N., Yamaji N., Ma J.F. (2008). Characterization of Substrate Specificity of a Rice Silicon Transporter, Lsi1. Pflug. Arch. Eur. J. Physiol..

[94] Coskun D., Deshmukh R., Shivaraj S.M., Isenring P., Bélanger R.R. (2021). Lsi2: A Black Box in Plant Silicon Transport. Plant Soil.

[95] Snyder G.H., Matichenkov V.V., Datnoff L.E. (2007). Silicon. Handbook of Plant Nutrition.

[96] Barreto R.F., Barão L., Prado R.M. (2023). Silicon: Transcellular and Apoplastic Absorption and Transport in the Xylem. Benefits of Silicon in the Nutrition of Plants.

[97] Mitani N., Chiba Y., Yamaji N., Ma J.F. (2009). Identification and Characterization of Maize and Barley Lsi2-Like Silicon Efflux Transporters Reveals a Distinct Silicon Uptake System from That in Rice. Plant Cell.

[98] Oliveira R.L.L., Prado R.M., Felisberto G., Checchio M.V., Gratão P.L. (2019). Silicon Mitigates Manganese Deficiency Stress by Regulating the Physiology and Activity of Antioxidant Enzymes in Sorghum Plants. J. Soil Sci. Plant Nutr..

[99] Moreira A.R., Fagan E.B., Martins K.V., Souza C.H.E. (2010). Resposta da cultura de soja a aplicação de silício foliar. Biosci. J..

[100] Frew A., Weston L.A., Reynolds O.L., Gurr G.M. (2018). The Role of Silicon in Plant Biology: A Paradigm Shift in Research Approach. Ann. Bot..

[101] Brunings A.M., Datnoff L.E., Ma J.F., Mitani N., Nagamura Y., Rathinasabapathi B., Kirst M. (2009). Differential Gene Expression of Rice in Response to Silicon and Rice Blast Fungus Magnaporthe Oryzae. Ann. Appl. Biol..

[102] Fauteux F., Rémus-Borel W., Menzies J.G., Bélanger R.R. (2005). Silicon and Plant Disease Resistance against Pathogenic Fungi. FEMS Microbiol. Lett..

[103] Frew A., Allsopp P.G., Gherlenda A.N., Johnson S.N. (2017). Increased Root Herbivory under Elevated Atmospheric Carbon Dioxide Concentrations Is Reversed by Silicon-Based Plant Defences. J. Appl. Ecol..

[104] Van Bockhaven J., Steppe K., Bauweraerts I., Kikuchi S., Asano T., Höfte M., De Vleesschauwer D. (2015). Primary Metabolism Plays a Central Role in Moulding Silicon-Inducible Brown Spot Resistance in Rice. Mol. Plant Pathol..

[105] Kolesnikov M.P., Gins V.K. (1999). Flavonoids and Silicon in Certain Plant Pollen. Chem. Nat. Compd..

[106] Manivannan A., Soundararajan P., Muneer S., Ko C.H., Jeong B.R. (2016). Silicon Mitigates Salinity Stress by Regulating the Physiology, Antioxidant Enzyme Activities, and Protein Expression in *Capsicum annuum* ‘Bugwang’. BioMed Res. Int..

[107] Silva E.S., Prado R.M., Santos D.M.M., Cruz F.J.R., Júnior de Almeida H., Campos C.N.S. (2015). Nitrogen Components, Growth and Gas Exchange in Spring Wheat Plants Grown under Interaction of Silicon (Si) and Nitrogen (N). Aust. J. Crop Sci..

[108] McCree K.J., Fernandez C.J. (1989). Simulation Model for Studying Physiological Water Stress Responses of Whole Plants. Crop Sci..

[109] Wu Y., Huang M., Warrington D.N. (2011). Growth and Transpiration of Maize and Winter Wheat in Response to Water Deficits in Pots and Plots. Environ. Exp. Bot..

[110] Herrero M.P., Johnson R.R. (1981). Drought Stress and Its Effects on Maize Reproductive Systems. Crop Sci..

[111] Tombeur F., Cooke J., Collard L., Cisse D., Saba F., Lefebvre D., Burgeon V., Nacro H.B., Cornelis J. (2021). Biochar Affects Silicification Patterns and Physical Traits of Rice Leaves Cultivated in a Desilicated Soil (*Ferric lixisol*). Plant Soil.

[112] Gong H.J., Chen K.M., Zhao Z.G., Chen G.C., Zhou W.J. (2008). Effects of Silicon on Defense of Wheat against Oxidative Stress under Drought at Different Developmental Stages. Biol. Plant..

[113] Amin M., Ahmad R., Ali A., Hussain I., Mahmood R., Aslam M., Lee D.J. (2018). Influence of Silicon Fertilization on Maize Performance Under Limited Water Supply. Silicon.

[114] Marschner H. (1995). Mineral Nutrition of Higher Plants.

[115] Bianchini H.C., Marques D.J. (2019). Tolerance to Hydric Stress on Cultivars of Silicon-Fertilized Corn Crops: Absorption and Water-Use Efficiency. Biosci. J..

[116] Datnoff L.E., Snyder G.H., Korndörfer G.H. (2001). Silicon in Agriculture.

[117] Taiz L., Zeiger E. (2010). Plant Physiology.

[118] Walters J.P., Archer D.W., Sassenrath G.F., Hendrickson J.R., Hanson J.D., Halloran J.M., Vadas P., Alarcon V.J. (2016). Exploring Agricultural Production Systems and Their Fundamental Components with System Dynamics Modelling. Ecol. Model..

[119] Flores R.A., Souza M.A.P., Andrade A.F., Bueno A.M., Abdala K.O., Souza Júnior J.P., Prado R.M., Santos G.G., Mesquita M. (2022). Does Foliar Application of Silicon under Natural Water Stress Conditions Increase Rice Yield in Subtropical Dry Regions?. Silicon.

[120] Flores R.A., Sousa M.A.P., Bueno A.M., Andrade A.F., Souza Júnior J.P., Abdala K.O., Prado R.M., Santos G.G., Mesquita M. (2021). Does Foliar Silicon Application Enhance the Biomass Yield of Millet Silage, and Does It Provide Significant Economic Gains?. Res. Soc. Dev..

[121] Freire A.H., Reis R.P., Fontes R.E., Veiga R.D. (2011). Eficiência econômica da cafeicultura no Sul de Minas Gerais: Uma aplicação da fronteira de produção. Coffee Sci..

[122] Alvares C.A., Stape J.L., Sentelhas P.C., Gonçalves J.L.M., Sparovek G. (2013). Köppen’s Climate Classification Map for Brazil. Meteorol. Z..

[123] Casaroli D., Rodrigues T.R., Martins A.P.B., Evangelista A.W.E., Alves Júnior J. (2018). Padrões de Chuva e de Evapotranspiração em Goiânia, GO. Rev. Bras. Meteorol..

[124] Santos H.G., Jacomine P.K.T., Anjos L.H.C., Oliveira V.A., Lumbreras J.F., Coelho M.R., Almeida J.A., Araújo Filho J.C., Lima H.N., Marques F.A. (2025). Sistema Brasileiro de Classificação de Solos.

[125] WRB World Reference Base for Soil Resources (2022). International Soil Classification System for Naming Soils and Creating Legends for Soil Maps.

[126] Teixeira P.C., Donagemma G.K., Fontana A., Teixeira W.G. (2017). Manual de Métodos de Análise de Solos.

[127] Kilmer V.J. (1965). Silicon. Methods of Soil Analysis.

[128] Allen R.G., Pereira L.S., Raes D., Smith M. (1998). Crop Evapotranspiration—Guidelines for Computing Crop Water Requirements—FAO Irrigation and Drainage Paper 56.

[129] Arruda F.B., Zullo Júnior J., Oliveira J.B. (1987). Soil Parameters for Calculating Available Water Based on Soil Texture. Rev. Bras. Ciência Solo.

[130] Pereira L.S., Valero J.A.J., Buendía M.R.P., Martín-Benito J.M.T. (2010). El Riego y Sus Tecnologías.

[131] Camargo F.A.O., Battisti R., Knapp F.M., Dalchiavon F.C. (2022). Maize Yield Gain Using Irrigation in the State of Rio Grande Do Sul, Brazil. Rev. Bras. Eng. Agríc. Ambient..

[132] Lima M.L. (2022). Adubação Foliar Com Silício na Soja e Milho de Segunda Safra. Ph.D. Thesis.

[133] Agranda Sementes Semente Milho Híbrido B2433 PWU. https://www.agranda.com.br/produto/milho-hibrido-b2433-pwu.

[134] Brevant Milho B2433PWU. https://www.brevant.com.br/produtos/milho/b2433pwu.html.

[135] Ministério da Agricultura, Pecuária e Abastecimento (2009). Regras Para Análise de Sementes.

[136] Sousa D.M.G., Lobato E. (2004). Cerrado: Correção do Solo e Adubação.

[137] Silva F.C. (2009). Manual de Análises Químicas de Solos, Plantas e Fertilizantes.

[138] Kraska J.E., Breitenbeck G.A. (2010). Simple, Robust Method for Quantifying Silicon in Plant Tissue. Commun. Soil Sci. Plant Anal..

[139] Noronha J.F. (1987). Projetos Agropecuários: Administração Financeira, Orçamento e Viabilidade Econômica.

[140] CEPEA Indicador do Milho ESALQ/BM&FBOVESPA. https://www.cepea.esalq.usp.br/br/indicador/milho.aspx.

[141] FGV Índice Geral de Preços. https://portalibre.fgv.br/igp.

[142] Fundação ABC Planilha de Custos de Mecanização Agrícola. https://fundacaoabc.org/wp-content/uploads/2019/11/Custo-de-Mecaniza%C3%A7%C3%A3o-MAIO2019.pdf.

[143] Barbosa J.C., Maldonado Júnior W. (2015). Experimentação Agronômica & AgroEstat—Sistema Para Análises Estatísticas de Ensaios Agronômicos.

